# Matrix Metalloproteinase 9 (MMP-9) and Interleukin-8 (IL-8) in Gingival Crevicular Fluid after Regenerative Therapy in Periodontal Intrabony Defects with and without Systemic Antibiotics—Randomized Clinical Trial

**DOI:** 10.3390/pathogens11101184

**Published:** 2022-10-14

**Authors:** Ewa Dolińska, Małgorzata Pietruska, Violetta Dymicka-Piekarska, Robert Milewski, Anton Sculean

**Affiliations:** 1 Department of Periodontal and Oral Mucosa Diseases, Medical University of Bialystok, ul. Waszyngtona 13, 15-269 Bialystok, Poland; 2 Department of Clinical Laboratory Diagnostics, Medical University of Bialystok, ul. Waszyngtona 15, 15-269 Bialystok, Poland; 3 Department of Statistics and Medical Informatics, Medical University of Bialystok, ul. Szpitalna 37, 15-295 Bialystok, Poland; 4 Department of Periodontology, Dental School University of Bern, Freiburgstrasse 7, 3010 Bern, Switzerland

**Keywords:** periodontitis, periodontal regeneration, interleukin 8, metalloproteinase 9, antibiotics, inflammatory markers, periodontal therapy, periodontal intrabony defects

## Abstract

The aim of our study was to assess changes in the levels of IL-8 and MMP-9 in gingival crevicular fluid (GCF) collected from the periodontal pocket before and after regenerative surgery with deproteinized bovine bone mineral (DBBM) and collagen membrane (GTR) either independently (DBBM/GTR) or with the postoperative administration of antibiotic (DBBM/GTR+AB). The study involved 41 patients, each with one intrabony defect. IL-8 and MMP-9 were determined before therapy and after 2 weeks, 4 weeks and 6 months following the surgical procedure by means of dedicated ELISA kits. No statistical differences were observed in the levels of IL-8 and MMP-9 after 2 weeks, 4 weeks and 6 months between the groups. The changes in the level of MMP-9 over time were not statistically significant in any group. The changes in the level of IL-8 were significant for the group given antibiotic but not in the nonantibiotic group in the follow-up period. IL-8 and MMP-9 were found to correlate positively but not after 4 weeks in the test group. Current assessment of IL-8 and MMP-9 obtained from GCF samples provides evidence that collagen matrix turnover occurs actively during the early healing phase in the periodontium after regenerative procedures. We observed positive correlations of MMP-9 and IL-8 throughout the study. However, we failed to reveal any differences regard parameters studied between the two groups.

## 1. Introduction

Although periodontitis is an infectious disease initiated by dental plaque microbiome, it is not the microorganisms themselves but the inflammatory reaction of the host to bacterial antigens that leads to the destruction of teeth-supporting tissues. Periodontitis occurs with episodes of tissue damage and periods of remission [1]. This process directly involves enzymes that belong to the family of matrix metalloproteinases (MMPs), mainly collagenases and gelatinases, degrading collagen in the soft tissues and bone [2], as well as numerous cytokines (including IL-8).

Extracellular matrix metalloproteinases (MMPs) are zinc-dependent endopeptidases. These enzymes are able to degrade basement membranes and extracellular matrix, thus enabling tissue remodeling and cell migration during physiological and inflammatory processes in the body. They are secreted by various cells of the host, such as polynuclear leukocytes, macrophages, fibroblasts, bone cells, epithelial and endothelial cells [3]. The major MMPs originating in neutrophils include MMP-8 (colagenase-2) and MMP-9 (gelatinase B). MMP-8 takes part in the degradation of interstitial collagen, whereas MMP-9, which is a gelatinase enzyme, degrades a few extracellular matrix proteins such as type IV collagen of the basal lamina. As these are major collagenolytic enzymes in saliva and GCF, they are considered responsible for collagen degradation in the course of gingivitis and periodontitis [4].

The activity of MMP-9 in GCF has been frequently studied, and its relationship with clinical periodontal parameters and progressive loss of clinical attachment has been identified [5,6]. It has also been reported that the level of MMP-9 in GCF and in periodontal ligament is increased in the course of periodontal disease and that it may be reduced by effective periodontal therapy [7,8]. Its role in bone resorption was revealed in an in vitro study [9]. It is therefore assumed that MMP-9, apart from soft tissue destruction, can play a key role in the initiation and progression of inflammatory bone damage in the course of periodontitis [10]. Taking the above into account, it can be suggested that MMP-9 is a regulator of periodontal lesions.

Il-8 is a potent chemoattractant targeting the recruitment and activation of neutrophilic granulocytes, and it is thus an indispensable mediator of inflammatory process. It can be secreted by various cells of the host, including monocytes/macrophages, lymphocytes, fibroblasts, and endothelial and epithelial cells [11]. Neutrophils themselves can be a source of IL-8 in response to LPS originating from periopathogenic bacteria [12]. Apart from bacterial endotoxins, IL-1, TNF-α and immunological complexes may also induce IL-8 secretion [13]. IL-8 exerts diverse effects on the activity and functions of neutrophils, i.e., it affects their adhesion to endothelial cells; shape change and transmigration chemotaxis; degranulation of primary and secondary granules containing a number of lysosomal enzymes (including primary MMP-9); and respiratory burst. IL-8 has appeared to be a very important mediator of the periodontal inflammatory process. A vital neutrophil-related role of IL-8 has been considered in the inflammatory destruction of tissues in the course of periodontitis [14].

The administration of systemic antibiotics with periodontal surgery is a routine procedure for preventing post-surgical complications and promote expected regeneration (clinical attachment level (CAL) gain) [15,16,17]. It is noteworthy that the number of infections after periodontal surgery is statistically low [18] and that the overuse of antibiotics generates many problems. The main is bacteria strain resistance. That is why precaution is recommended regarding the frequent use of antibiotics. Additionally, there is a concern regarding antibiotic side effects such as gastrointestinal disorders, pseudomembranous enterocollitis and superinfections [19,20]. Systemic administration of antibiotic may affect the composition of GCF and modulate the course of the inflammatory response.

Literature provides little information on the relationship between IL-8 and MMP-9 in gingival crevicular fluid associated with vertical bone loss. No data are available regarding changes in the levels of IL-8 and MMP-9 at the sites after regenerative procedures. 

The aim of our study was to assess changes in the levels of IL-8 and MMP-9 in GCF collected before regenerative procedure (using deproteinized bovine bone mineral (DBBM) and collagen membrane either independently or with postoperative antibiotic) and after 2 weeks, 4-weeks and 6 months. We also evaluated alterations in the volume of GCF at the site after regenerative therapy in both groups in an annual follow-up. 

## 2. Materials and Methods

### 2.1. Study Population and Experimental Design

The study was performed according to the Helsinki Declaration after previous acceptance obtained from the bioethical committee (Bioethical Committee, Medical University of Bialystok, R-I-002-302-2013). The study accomplished the CONSORT guidelines for randomized trial. A total of 41 generally healthy adults were recruited to the study (27 females and 14 males, mean age 41.8). The inclusion criterion was the presence of at least one intrabony defect that met the following conditions: probing depth PD ≥ 6mm, defect depth in radiography (straight angle technique) greater or equal RxD ≥ 3mm, defect width greater or equal RxW ≥ 2mm. Other criteria for patients’ enrolment included written consent to participate in the study, age 18+, no allergy to penicillin, good oral hygiene (full mouth plaque score FMPS < 20%, full mouth bleeding on probing FMBOP < 20%), and no addiction to smoking. 

Subjects who had taken antibiotics at least 3 months prior to enrolment or suffering from general diseases that might affect healing, as well as pregnant and lactating women, were excluded. 

### 2.2. Surgical Procedures

Patients who were enrolled in the study were allocated to the test group (DBBM/GTR+AB) or the control group (DBBM/GTR) based on a computer-generated randomization list (generated by R.M using a self-written programme). All the patients underwent guided tissue-regenerative procedures (GTR) combined with xenogeneic materials (Collprotect® and Cerabone®, respectively, botiss, biomaterials GmbH, Zossen, Germany) following the same surgical procedure as published previously [21]. The only difference between patients randomized to the test group and the control group was the systemic antibiotic therapy in the test group. The tested patients received amoxicillin (Ospamox, Sandoz GmbH, Kundl, Austria) on the day of the procedure and followed by 2 × 1 g/day for 7 days. Postoperatively patients were instructed to rinse mouth twice a day with 0.2% chlorhexidine digluconate solution (Eludril, Pierre Fabre Laboratories, Paris, France). After suture removal (2 weeks post-op), patients resumed brushing at the operated area using an ultrasoft post-surgical brush.

### 2.3. Clinical Examinations and Postoperative Care

All the clinical examinations were performed by an experienced calibrated periodontist (ED). Clinical parameters were evaluated before therapy and 6 and 12 months after the procedure and involved measurements of probing depth (PD) and gingival recession height (GR). CAL was calculated mathematically. A periodontal probe calibrated every 1 mm was used for clinical measurements (PCPUNC 15, Hu-Friedy, Chicago, IL, USA). Only the deepest measurement determined in the preoperative examination was used for statistical analysis. The measurements were rounded up to full millimetre. Full mouth plaque score (FMPS) was analysed separately as the percentage on all dental surfaces (4 surfaces for each tooth) [22], just as was full mouth bleeding on probing (FMBOP). 

The patients from the test group (DBBM/GTR+AB) were given amoxicillin (Ospamox, Sandoz GmbH, Kundl, Austria) on the day of the surgery for 7 days at a dose of 1g every 12 h. After the procedure, all the patients were recommended to rinse the mouth with 0.2% solution of chlorhexidine digluconate (Eludril, Pierre Fabre Laboratories, Paris, France) for 2 weeks until removal of stitches. Throughout one-year follow-up, patients from both groups received scrupulous periodontal care. Check-up visits were scheduled 1, 2, 4 weeks after the procedure. During these visits, oral hygiene was monitored, and the surgical area was polished. Next, supragingival scaling was performed 2, 3, 6 and 12 months after the surgery, apart from oral hygiene and bleeding control (FMPS, FMBS, FMBOP). The patients were encouraged to maintain ideal oral hygiene. On every check-up visit, GCF was collected from the deepest pocket corresponding to the intrabony defect that had been surgically treated. The patients were well motivated. All the check-up visits were performed. No patient resigned from participation in the study. 

### 2.4. GCF Sampling

At baseline and on every check-up visit, GCF was collected from the periodontal pocket corresponding to the regenerated intrabony defect, and the sulcus fluid flow rate (SFFR) was determined in relative Periotron-units (PU). The tooth was isolated with cotton rolls, dental plaque was gently removed and the tooth was air dried. GCF was collected using paper strips (Periopaper, Interstate Drug Exchange, Amityville, NY, USA), which were placed in the periodontal pocket at 1–2 mm depth for 30 s. The blood-contaminated strips were discarded. The GCF volume absorbed on a paper strip (Sulcus Fluid Flow Rate- SFFR) was measured using a calibrated device (Periotron 8000, Oraflow, Plainview, NY, USA) and expressed in Periotron Units (PU). After measurement, the samples were immediately placed in Eppendorf tubes containing 200 μL phosphate buffered saline (PBS) and frozen.

### 2.5. GCF IL-8 and MMP-9 Analysis

The gingival crevicular fluid (GCF) samples collected at baseline and after 2 weeks, 4 weeks and 6 months were used for laboratory analysis. Concentrations of MMP-9 and IL-8 were measured in cell culture supernatant using an enzyme-linked immunosorbent assay (ELISA), Quantikine Human MMP-9 (Cat. number: DMP900) and Human IL-8/CXCL8 (Catalog number D8000C); (R&D Systems, Inc. Minneapolis, MN, USA) according to manufacturer’s instruction. The polyclonal human MMP-9 and IL-8 antibodies were pre-coated onto 96-well strip plates. Diluted human samples and standards of known MMP-9 and IL-8 concentrations were added to the wells. The biotinylated human MMP-9 and IL-8 polyclonal antibodies were added. These detection antibodies bind to the antigen, thus completing the sandwich. After the next step, washing, an enzyme was added that bound to the second antibody. The peroxidase substrate was added to induce the coloured reaction product. The intensity of this coloured product was directly proportional to the concentration of the particular MMP-9 and IL-8 present in the sample. Reading of the absorbance was performed on a microplatelet reader Multiscan Go (Thermo Fisher Scientific, Waltham, MA, USA).

According to protocol, MMP-9 samples were 100-fold diluted prior to analysis using the assay diluent (Calibrator Diluent RD5-10). The manufacturer of assay kits referred to the intra-assay coefficient of variation (CV%) as 2.0 % at MMP-9 mean concentration 0.833 ng/mL, SD = 0.0017 ng/mL. The manufacturer of assay kits referred to the intra-assay coefficient of variation (CV%) as 4.6 % at IL-8 mean concentration 115 pg/mL, SD = 5.3 ng/mL.

The results were counted as the amount per 30 s per measure point and expressed in pg/mL for IL-8 and ng/ml for MMP-9. Lamster et al. [23] stated that depicting data as a concentration might not be appropriate for GCF as this assumes that each sample contains an equal volume of fluid that is representative of the total fluid volume. Reporting total mediator content per 30 s sample removes a potential source of error from the analysis [24].

### 2.6. Statistical Analysis

In the statistical analysis, the normality of the distribution was verified using the Kolgomorov–Smirnov tests with Lillefors correction and the Shapiro–Wilk test. The distribution normality of the analysed quantitative variables was not found. To compare the quantitative variables without distribution normality the nonparametric Mann–Whitney U test was applied for the two groups. The Friedman test was used to compare dependent variables in the case of many variables. Spearman’s rank correlation coefficient was also determined. 

The results were considered statistically significant for *p* < 0.05. For calculations, the (Statistica 13.3, Tibco Software Inc., Palo Alto, CA, USA), was employed.

## 3. Results

The test group characteristics and the clinical, radiological and intraoperative findings with reentry were published earlier [21].

**Figure 1:**
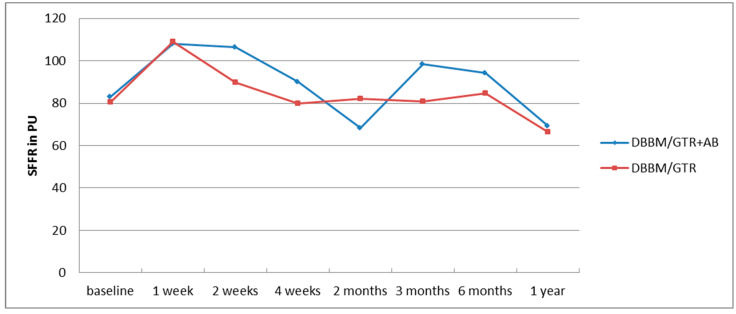
Changes in the mean GCF volume (SFFR) in the annual follow-up after regenerative surgery in the test (DBBM/GTR+AB) and control (DBBM/GTR) group.

Prior to treatment, the levels of SFFR, MMP-9 and IL-8 in GCF did not show statistically significant differences between the two groups. No statistical differences were also found in the levels of IL-8 and MMP-9 after 2 weeks, 4 weeks and 6 months between the test group and the control group. The GCF volume (SFFR) also did not differ between the groups at any time point. The changes in the mean volume of the gingival crevicular fluid over time in the two groups have been presented in Figure 1. 

**Figure 2:**
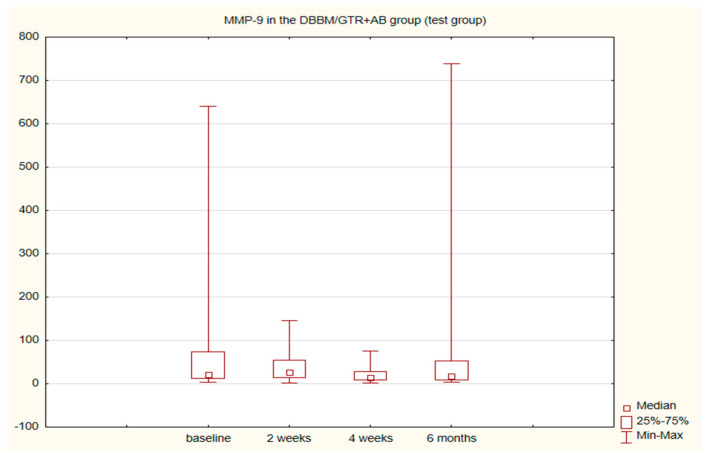
Box plot of changes in the MMP-9 amount in the six-month follow-up in the test (DBBM/GTR+AB) group expressed in ng/mL per 30 s sample.

**Figure 3:**
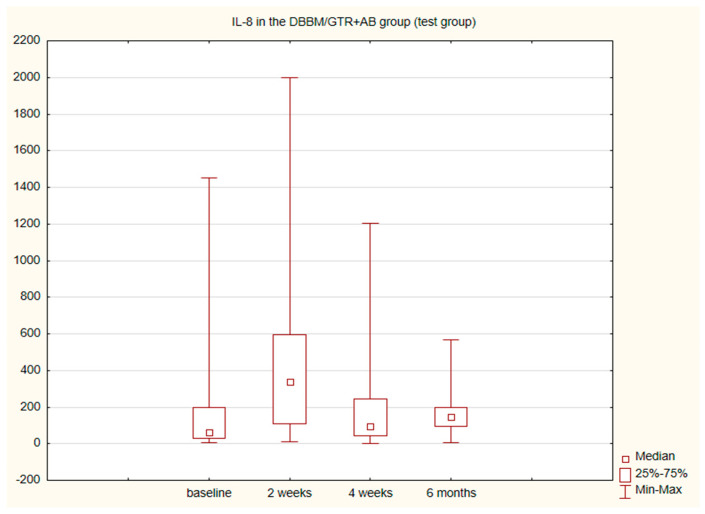
Box plot of the changes in the IL-8 amount in the six-month follow-up in the test (DBBM/GTR+AB) group expressed in pg/mL per 30 s sample.

**Figure 4:**
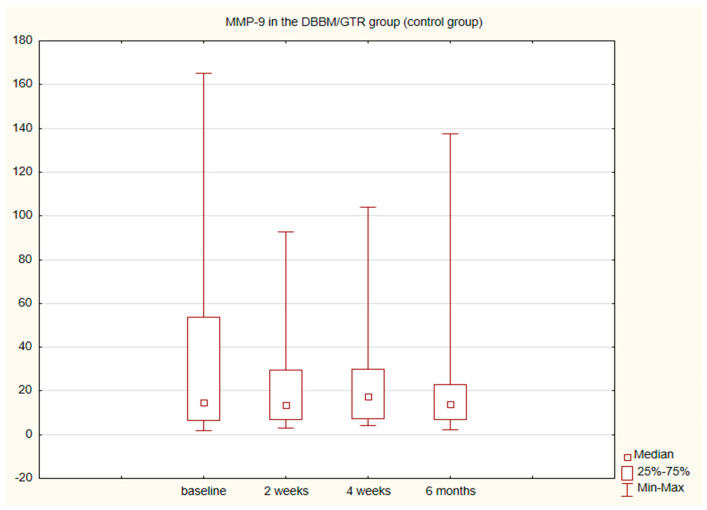
Box plot of the changes in the MMP-9 amount in the six-month follow-up in the control (DBBM/GTR) expressed in ng/mL per 30 s sample.

**Figure 5:**
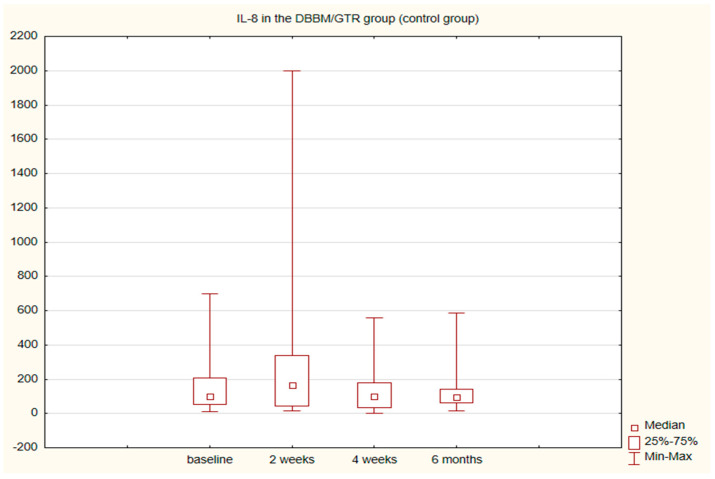
Box plot of the changes in the IL-8 amount in the six-month follow-up in the control (DBBM/GTR) group expressed in pg/mL per 30 s sample.

The alterations in the level of MMP-9 over time were not statistically significant in any of the groups. The level of IL-8, just like the change in the GCF volume (SFFR) over time, was significant in the antibiotic group but not in the nonantibiotic group. Changes in the levels of MMP-9 and IL-8 are shown in Table 1 and Table 2. Since nonparametric tests were used for analysis, the medians and the minimum and maximum levels of MMP-9 and IL-8 are depicted in box plots (Figure 2, Figure 3, Figure 4 and Figure 5).

The analysis of the Spearman’s correlation rank showed relationships between the levels of MMP-9 and IL-8 in both groups at all time points except for the 4-week point in the DBBM/GTR+AB group. The correlations found were positive and the relationships relatively strong and moderate. The power of the respective correlations are presented in Table 3. 

## 4. Discussion

Clinical and radiological examinations remain the golden standard in the assessment of intrabony periodontal defects before and after surgical treatment. However, the elucidation of differences in the inflammatory profile of the sites before and after regenerative procedures using collagen membranes and DBBM with and without additional antibiotic enriches the knowledge of periodontal disease, including its course in periodontal defects of the bone and its healing in surgical sites. We examined the levels of MMP-9 and IL-8 in periodontal pockets associated with vertical bone defects prior to treatment, and 2 weeks, 4 weeks, 2 months, 3 months, 6 months and 12 months after regenerative procedure. The examination scheduled in this way allowed qualitative assessment (MMP-9, IL-8) and qualitative evaluation (GCF volume) of the fluid from the sites subjected to regenerative procedures.

The GCF itself is a plasma filtrate or inflammatory exudate and it can be obtained from the gingival crevice that surrounds the tooth. Thus, its components originate both from the host and from microorganisms residing there. The former include inflammatory markers such as enzymes, cytokines and interleukins [25]. The relationship between the increased volume of GCF and greater severity of periodontitis has been well documented [26,27,28]. It is suggested that the elevated volume of GCF and bleeding after probing are one of the earliest signs of inflammation and the increase in GCF volume itself can manifest subclinical inflammation [29]. In our study, the mean volume of the fluid collected from the pocket within 30 s (SFFR) was found to increase one week after the surgery and remained high 2 weeks following the procedure in both groups. This indicates the enhancement of inflammation during early surgical wound healing. After one month, 2-, 3- and 6 months the GCF volume remained close to the initial values in the nonantibiotic group and showed a marked decrease after 2 months in the DBBM/GTR+AB group. In the one-year follow-up in both groups the mean amount of GCF decreased below that found at baseline. This can be associated with shallowing of the pockets and limitation of inflammation at the site of fluid collection [21]. However, only in the antibiotic group the changes in the fluid volume were statistically significant.

In very few studies concerning the regenerative procedures using collagen membranes (GTR), inflammatory markers have been additionally determined in GCF. After such procedures, platelet-derived growth factor-BB (PDGF-BB) [30,31], PDGF-AB, osteoprotegerin (OPG), vascular endothelial growth factor (VEGF) [32] and transforming growth factor β1 (TGF-β1) [33] have been assessed. Pellegrini et al. using the same GCF collecting tools as in our study (Periopaper, InterstateDrug Exchange, Amityville, NY) evaluated local biomarkers of healing in sites after regenerative procedures and flap surgeries. The fluid for analysis was collected 3–5 days and 1, 2 and 3 weeks after surgery. The immunological profiles of E-cadherin, EGF (epidermal growth factor), TGF-β1, VEGF, FGF-2 (fibroblast growth factor 2), MMP-1 (matrix metalloproteinase 1), TIMP-1 (tissue inhibitor metalloproteinase 1), BMP-7 (bone morphogenetic protein 7) and OPG were determined. Within 3 weeks after surgical procedures no significant differences were noted in the profile of the majority of the molecules assessed. It was only found that BMP-7 increases in patients that respond well to therapy and decreases in those with worse response, whereas MMP-1 grows in poorly responding and does not change in well responding patients. Of many biomolecules studied, these two were found to be the most accurate predictors of a beneficial clinical outcome [34]. Metalloproteinases 1 and 8 and TIMP-1 were also investigated by Okuda et al. after surgical procedures using EMD (enamel matrix derivative) in intrabony defects 2, 4 and 12 weeks after surgery. The use of EMD was found to promote healing as compared to placebo. After 2 weeks in both groups the immunological parameters increased significantly, which was associated with more severe inflammation directly after surgery. In the EMD group, the values of TIMP-1, MMP-1 and -8 were found to rapidly return to the initial levels [35]. To the best of our knowledge, no researcher has investigated MMP-9 after regenerative procedures, although its role in the pathogenesis of periodontal disease [36] and in alveolar bone resorption is widely described [10,37]. We observed no statistical differences in the amount of MMP-9 prior to and after regenerative procedures in any of the study groups. We also observed no differences between the groups before or after surgery in the time points studied. 

Sijari et al. assessed IL-8 in GCF after resective surgery. The examination was performed prior to and 14 days after surgical procedure and showed a reduction in the level of IL-8 after 2 weeks [38]. This is not in agreement with our findings showing that after 2 weeks the amount of IL-8 increases statistically significantly in the antibiotic group and insignificantly in the nonantibiotic group. After 4 weeks and 6 months, the amount of IL-8 was close to the baseline value. We did not observe any differences in the amount of IL-8 between the groups. 

The main chemoattractant for neutrophils, i.e. IL-8 (CXCL8), is positively conjugated with MMP-9. MMP-9 has been reported to cleave IL-8 molecule removing 6 out of 77 amino acids at its amino terminal, giving rise to IL-8 (7-77), which is a more potent chemoattractant than the initial form. This causes enhanced recruitment of neutrophils at the inflammation site and thus more intense secretion of MMP-9 [39]. In our study, the amount of secreted IL-8 also correlated positively, moderately or strongly, with MMP-9 in both groups of patients in all time points studied except for that after 4 weeks in the DBBM/GTR+AB group. This seems to indicate that in the regenerated intrabony defects the increase in the amount IL-8 involves a rise in the level of MMP-9. The similar relationship was observed in lower genital tract of women, where the IL-8 cleaving activity significantly correlated with active MMP-9. It suggests that MMP-9 can cleave IL-8 at mucosal sites [40]. Additionally, there are several bacterial proteases that influence IL-8. One of the best studied is *Porphyromonas gingivalis,* a periodontopathogen that produces proteases that can cleave IL-8 [41,42]. Gingipains are endopeptidases responsible for proteolitic activity of *P. gingivalis* and are able to convert IL-8 to a more potent species followed by slow degradation of chemokine biological activity. As well they seem to play main role in the evasion of host defence mechanisms [42,43].

A systematic review of 2020 confirms the scarcity of data concerning the expression of markers of angiogenesis, regeneration and inflammation in the GCF at the stage of early and late healing after surgical interventions in vertical defects [44]. Therefore, further studies with consistent methodology are necessary, and our research meets this need. Recently, a work concerning the molecular profiling of intrabony defects was published. Intrabony defects presented significantly increased GCF volume as well as increased IL-1 alpha, IL-1 beta, IL-6, IFN gamma and MMP-8 levels compared to periodontally healthy sites [45]. The use of local biomarkers offer the possibility for real-time assessment of the healing process. Knowledge of molecular events in the early wound healing will enable modulating the expression profile of biomarkers thereby to improve clinical outcomes of periodontal regeneration.

We understand that IL-8 and MMP-9 are two of many biological mediators involved in wound healing. Several other biological factors should be studied to better understand different phases of healing. Another limitation of our study is that it was powered for clinical outcomes, but there is no similar study concerning vertical defects and Il-8 and MMP-9 amount in GCF before and after periodontal regeneration. Maybe larger population group would be necessary to make definitive conclusion.

## 5. Conclusions

To sum up the amount of GCF one and two weeks after surgical procedures increased. It indicates development of inflammation in early stage after surgery. The results also show early healing in periodontal intrabony defects through the prism of elevated levels of IL-8 two weeks after regeneration and positive correlations between MMP-9 and IL-8 throughout the study. We failed to find differences in the immunological parameters between the group with and without antibiotic therapy.

## Figures and Tables

**Table 1:** Changes in MMP-9 in the six-month follow-up in the test (DBBM/GTR+AB) and control (DBBM/GTR) groups expressed in ng/mL per 30 s sample.

	DBBM/GTR+AB Me (Q_1_; Q_3_)	DBBM/GTR Me (Q_1_; Q_3_)	p** (Group vs. Group)
baseline	20.85 (12.08; 73.16)	14.76 (6.46; 53.63)	NS
2 weeks	26.35 (13.34; 55.1)	13.37 (7.03; 29.67)	NS
4 weeks	14.7 (8.49; 28.32)	17.52 (7.32; 29.78)	NS
6 months	18.14 (9.45; 52.99)	14.13 (6.92; 23.07)	NS
p* (time changes)	NS	NS	

Me—median, Q—quartile, p*—Friedman’s ANOVA for multiple comparisons, p**—Mann–Whitney U test, NS—nonsignificant.

**Table 2:** Changes in the IL-8 amount in the six-month follow-up in the test (DBBM/GTR+AB) and control (DBBM/GTR) groups expressed in pg/mL per 30 s sample.

	DBBM/GTR+AB Me (Q_1_; Q_3_)	DBBM/GTR Me (Q_1_; Q_3_)	p** (Gruop vs. Group)
baseline	64.42 (28.34; 199.6)	99.7 (51.76; 208.3)	NS
2 weeks	337.5 (107.7; 593.8)	163.9 (42.37; 336.75)	NS
4 weeks	95.21 (45.35; 246.6)	98.68 (34.98; 180,5)	NS
6 months	146.7 (96.32; 198.6)	95.53 (60.36; 142.7)	NS
p* (time changes)	*p* = 0.01	NS	

Me—median, Q—quartile, p*— Friedman’s ANOVA for multiple comparisons, p**—Mann–Whitney U test, NS—nonsignificant.

**Table 3:** Correlations (Spearman test) between GCF IL-8 and MMP-9 levels in the 6-month follow-up in the test (DBBM/GTR+AB) and control (DBBM/GTR) groups in the regenerated area.

IL-8 & MMP-9	DBBM/GTR+AB		DBBM/GTR	
	R	*p*	R	*p*
baseline	0.58	0.006	0.55	0.013
2 weeks	0.43	0.049	0.67	0.001
4 weeks	0.24	0.3 (NS)	0.65	0.002
6 months	0.86	0.000001	0.7	0.0006

NS—non significant.

## Data Availability

The datasets used and/or analysed during the current study are available from the corresponding author on reasonable request.
